# Cytoplasmic and Nuclear Effects on Agronomic Traits in Diploid Interspecific Potato Hybrids

**DOI:** 10.3390/ijms262210841

**Published:** 2025-11-08

**Authors:** Paulina Smyda-Dajmund, Alicja Macko-Podgórni, Dorota Sołtys-Kalina

**Affiliations:** 1 Plant Breeding and Acclimatization Institute—National Research Institute in Radzików, Młochów Division, Platanowa Str. 19, 05-831 Młochów, Poland; d.soltys@ihar.edu.pl; 2 Department of Plant Biology and Biotechnology, Faculty of Biotechnology and Horticulture, University of Agriculture in Krakow, Al. 29 Listopada, 31-425 Krakow, Poland; a.macko@urk.edu.pl

**Keywords:** cytoplasm, *Solanum tuberosum*, 2C-value, organelle DNA content, breeding traits

## Abstract

The cultivated potato (*Solanum tuberosum* L.) is a globally important crop with a narrow genetic pool, making it vulnerable to biotic and abiotic stresses. The present study analyzed the relative content of the nuclear, mitochondrial, and plastid genomes and their contributions to agronomic traits in 30 diploid interspecific potato hybrids with diverse cytoplasmic types and pedigrees. The nuclear genome size (2C-value) was estimated using flow cytometry, while the organelle DNA content and cytoplasm types were determined by quantitative polymerase chain reaction (qPCR) and multiplex PCR, respectively. The genome size of individual diploid genotypes remained stable across cultivation conditions, such as in vitro or greenhouse environments. Significant variation was observed in genome size, organelle content, and cytoplasmic types, which were associated with differences in pollen fertility and starch content. Kendall’s correlation analysis revealed a strong positive correlation between the content of plastid and mitochondrial DNA, and between starch content and chip colour after cold storage. Principal component analysis (PCA) demonstrated that variation in plastid and mitochondrial DNA content explained differences among genotypes, with nuclear DNA content contributing independently. Notably, cytoplasmic male sterility was observed in some T-type cytoplasm genotypes, thus highlighting the role of nuclear–cytoplasmic interactions. The results obtained demonstrate that organelle genome composition exerts a significant influence on agronomic traits and offer valuable insights into the potential for the enhancement of potato breeding programmes through the analysis of cytoplasm and nuclear genomes.

## 1. Introduction

The cultivated potato (*Solanum tuberosum* subsp. *tuberosum* L.) is one of the most widely grown crops globally, ranking fourth in terms of food production after rice (*Oryza sativa* L.), wheat (*Triticum aestivum* L.), and maize (*Zea mays* L.). The potato belongs to the *Solanaceae* family, which comprises numerous other important agricultural species, including the tomato (*S. lycopersicum* L.), the eggplant (*S. melongena* L.), the pepper (*Capsicum annuum* L.), and the tobacco (*Nicotiana tabacum* L.). The potato was introduced to regions outside the Andes at the end of the sixteenth century [1]. The species was introduced to Europe from a limited gene pool and exhibits narrow genetic diversity, making it susceptible to various biotic and abiotic stresses. This poses a significant challenge for modern potato breeding, which aims to improve the quality and resistance traits of the potato. The utilization of wild potato relatives and diploid intraspecific potato hybrids in the development of new cultivars provides a source of germplasm that carries resistance to biotic and abiotic stresses.

The potato possesses three cellular organelles that contain DNA: the nucleus, plastids, and mitochondria. Coordinated interactions between nuclear and organellar genomes ensure proper plant function [2]. The nuclear genome is the primary source of genetic control over the inheritance of the majority of phenotypic traits. However, cytoplasmic determinants and the coordinated interaction between the nuclear and organellar genomes have been showed to significantly influence particular physiological pathways [3]. It is evident that the vast majority of proteins and regulatory elements required for organelle biogenesis, as well as for the transcriptional and translational processes, and structural organization of the mitochondrial and chloroplast genomes, are encoded by nuclear loci and subsequently imported into the respective organelles. Conversely, organelles have been demonstrated to possess the capacity to transmit regulatory signals to the nucleus, thereby modulating the expression of nuclear genes through a mechanism known as retrograde signalling [4]. An example of a protein involved in retrograde signalling between the plastid and nucleus is GUN1, a plastid-localized member of the pentatricopeptide repeat (PPR) protein family. GUN1 has been determined to be a pivotal integrator of plastid-to-nucleus signals during chloroplast biogenesis, through its RNA-binding PPR-SMR structure. By influencing developmental checkpoints in plastid differentiation, it modulates nuclear gene expression in response to plastid status [5]. Another example is the mitochondrial ATP synthase beta-subunit (ATPB), a nuclear-encoded component of the mitochondrial inner membrane ATP synthase complex. Reduced ATP synthase activity has been shown to affect not only mitochondrial signalling but also plastid signalling networks, thereby influencing nuclear gene regulation associated with both organelles [6]. Environmental stresses such as high light, drought, heat, or nutrient deficiency disturb organelle homeostasis and activate retrograde signalling pathways [7]. These stress-induced signals such as reactive oxygen species (ROS) alter nuclear gene expression via transcription factors like ABI4, which coordinate organelle function and cellular stress responses. Although nuclear–cytoplasmic signalling and plastid–mitochondrial crosstalk have been repeatedly identified, the exact functional relationship between those systems remains incompletely understood. Understanding the sequence of potato genomes, their interactions, and their influence on the development of agronomic traits would open up new possibilities for potato improvement.

The diploid potato genomes DM [8], M6 [9], RH [10], and ‘Solyntus’ [11] have been sequenced. In 2011, the nuclear genome of the homozygous diploid line (double haploid) *S. phureja* DM1-3 516 R44 was sequenced by the Potato Genome Sequencing Consortium and became the most widely used potato reference genome. The haploid nuclear genome of the DM potato comprises 12 chromosomes, and its size is estimated to be approximately 840 Mb. It contains 39,031 protein-coding genes [8].

The potato is characterized by a medium-sized genome in comparison to other crop plants [12]. The smallest plant genome, with a size of approximately 61 Mb/1C, has been identified in the species *Genlisea tuberosa*, a carnivorous angiosperm endemic to Brazil [13]. The largest genome of about 149,000 Mb/1C of DNA has been found to belong to the angiosperm *Paris japonica*, a species of rare plant from the Melanthiaceae family, endemic to the island of Honshu in Japan [13]. Many potato lines and cultivars are tetraploids (2*n* = 4*x* = 48), which means that they possess four sets of haploid chromosomes [14]. The autotetraploid genome of *S. tuberosum* L. is characterized by high levels of heterozygosity and suffers from strong inbreeding depression.

In the potato, the cytoplasm (including the plastid and mitochondria) is maternally inherited [15] and is involved in several biological processes, including energy metabolism, photorespiration, photosynthesis, amino acid biosynthesis, coenzyme biosynthesis, and programmed cell death [16]. Multiple copies of plastid and mitochondrial DNA exist with one copy of nuclear DNA. The potato cytoplasm was first divided into five cytoplasmic types: T/β, W/α, W/γ, W/δ, A/ε, and S/ε [17]. Hosaka and Sanetomo [18] distinguished six potato cytoplasm types: W (W/α, W/β, W/γ), T (T/β), D (W/α), A (A/ε), P (S/ε), and M (C/ε) based on six molecular markers, specific to mitochondrial and chloroplast DNA, amplified in a multiplex PCR reaction [18]. Cytoplasm types play a crucial role in the development of several agronomic traits in potato; e.g., W/γ-type cytoplasm is associated with higher tuber starch content and functional male sterility [19]. The T-type cytoplasm in *S. tuberosum* subsp. *tuberosum* is characterized by a higher tuberization, tuber yield, and greater tuber numbers.

The potato mitochondrial genome (mitogenome) is characterized by its multichromosomal nature, exhibiting both linear and circular DNA molecules [20]. The quantity of autonomous molecules present within the potato mitogenome exhibits variation among different potato genotypes. Cultivars such as Cicero and Désirée possess a mitogenome of a total size of 474,520 bp, consisting of two 49,229 bp and 112,797 bp autonomous circular molecules and one linear sequence of 312,491 bp [20]. The mitogenome structure of diploid potato interspecific hybrids also indicates complexity. The nine potato diploids were divided into three groups according to the number of mitogenome molecules and their structure [21]. Three groups were identified, each containing one linear form and three or four circular molecules. Most of the mitogenomes were similar in sequence. An exception was the mitogenome of *S. okadae*, which shows different molecular arrangements [21]. The analysis of mitogenome sequences from 13 potato accessions of various taxa has revealed the presence of three independent circular molecules. Only the mitogenome of *S. bukasovii* had a single circular molecule [22].

Potato mitochondrial genomes have been found to contain a set of conserved genes, which encode NADH dehydrogenases, ATP synthases, ribosomal proteins, cytochrome C, cytochrome c oxidases, succinate dehydrogenases, cytochrome c reductase, maturase, and transport membrane protein [20,21]. One ribosomal protein gene (*rps14*) appeared to be a pseudogene with its functional copy in the nucleus [23]. The presence of fragments of plastid DNA, integrated into the mitochondrial genome, has also been observed, which may indicate recombination between organelles. In total, approximately 2000 different proteins were identified that were found in the structure of the mitochondria [24].

The chloroplast DNA (plastome) of the potato is characterized by a higher degree of structural and gene content conservation than the nuclear and mitochondrial DNA [22]. The potato plastome ranges in size from 154 kb to 156 kb, and it is composed of a single circular DNA molecule. Its structure is divided into three regions: a large single-copy region (LSC) of a size of about 86 kb, a small single-copy region (SSC) of about 18 kb, and two inverted repeat regions (IRa and IRb) of about 25 kb. The variability of the plastome results from insertions and deletions, single-nucleotide polymorphism, or polymorphic single-sequence repeats [25]. Between 3000 and 4000 proteins have been identified in the chloroplast structure [26]. Most of these are encoded in the nucleus and transported to organelles.

In this study, 30 diploid interspecific potato hybrids were analyzed for their genomic DNA content, mitochondrial and plastid content, and cytoplasm type. Those hybrids have been previously characterized in terms of important agronomic traits such as tuber starch content, pollen fertility, presence of 2n gametes, chip colour after harvest, and cold storage. Relationships were investigated between the genome content and phenotypic traits. The diploid hybrids analyzed possess diverse cytoplasmic types and varying levels of male fertility, and they exhibit traits valuable for modern breeding. Consequently, they represent promising genetic resources for broadening the genetic base of cultivated potato.

## 2. Results and Discussion

The analysis of nuclear DNA content (2C-value) among the studied diploid potato hybrids revealed variation, with values ranging from 1.680 pg in DW 82-648 to 1.833 pg in DG 9 (Supplementary Table S1). The highest DNA content observed in DG 9 may suggest the presence of repetitive sequences or structural variation, whereas the lowest value in DW 82-648 may reflect a more compact genome organization. Both genotypes originate from *S. chacoense* and *S. tuberosum*. Additionally, DG 9 contains *S. yungasense*, and DW 82-648 contains *S. gourlayi* in its pedigree. The genome size in pg was also determined from leaves using flow cytometry by [27] for selected diploid potato species. The genome size varied from 1.14 pg for *S. bulbocastanum* to 1.38 pg for *S. tuberosum* cv. Zel 1136. Variation in nuclear DNA content among hybrids and diploids may result from historical introgressions, chromosomal rearrangements, or hybridization events and could have downstream implications for developmental processes and agronomic performance [28]. It is noteworthy that, although the observed variation is moderate, even subtle changes in genome size can be indicative of underlying genomic events, such as segmental duplications, transposon activity, or deletions, particularly in hybrid material derived from wide crosses [28]. We found that the genome size of individual selected diploid genotypes DG 08-28/13, DG 00-683, and DG 08-305 do not change depending on growing conditions, such as in vitro, post-in vitro planting, or first tuber generation in greenhouse environments (Table 1).

The relative number of plastid and mitochondrial copies to the nuclear genome varied among the analyzed diploid forms. The range of results obtained for pt/nuc DNA was 176.7–10,384.8 of relative plastid copy numbers, while for mt/nuc DNA, this value ranged from 17.4 to 702.7 (Supplementary Table S2). Characterized genotypes exhibit four types of cytoplasm: T, D, W, and P. Of the 30 genotypes analyzed, 19 had cytoplasm type T, 5 had type D, and 3 each had types W and P (Supplementary Table S2). This proportion of individual cytoplasm types is associated with the directional selection of genotypes that exhibited better agronomic traits (e.g., higher starch content) and the selection of parental forms as mothers in crosses in order to avoid male sterility. Other agronomic characteristics are described in [29]. In brief, the tuber starch content of the analyzed genotypes was diversified. High-starch genotypes (a starch content above 15%) predominated. Among these genotypes, the average starch content ranged from 15.3% for DG 88-89 to 29% for DG 83-2025 [26]. The starch content among low-starch genotypes was between 12.6% (DG 08-28/13) and 14.7% (DG 88-215; DG 03-226) [29]. Pollen fertility and 2n gamete content varied among the genotypes/forms. Fertility ranged from 0 to 90% of stained pollen grains [29]. All genotypes, except for male-sterile 90 HAE/35, showed fertility above 30%, which means that these forms can be successfully used in crossing programmes. The colour of the chips after harvest ranged from 3.4 to 8.5, while for CS, it ranged from 2.2 to 8.5.

The Shapiro–Wilk test was used to assess the normality of the phenotypic data distribution (Table 2). The results indicated that chip colour after cold storage (CS) follows a normal distribution (*p* = 0.1377). However, all other evaluated traits significantly deviated from normality at the *p* < 0.05 threshold (Table 2).

**Figure 1:**
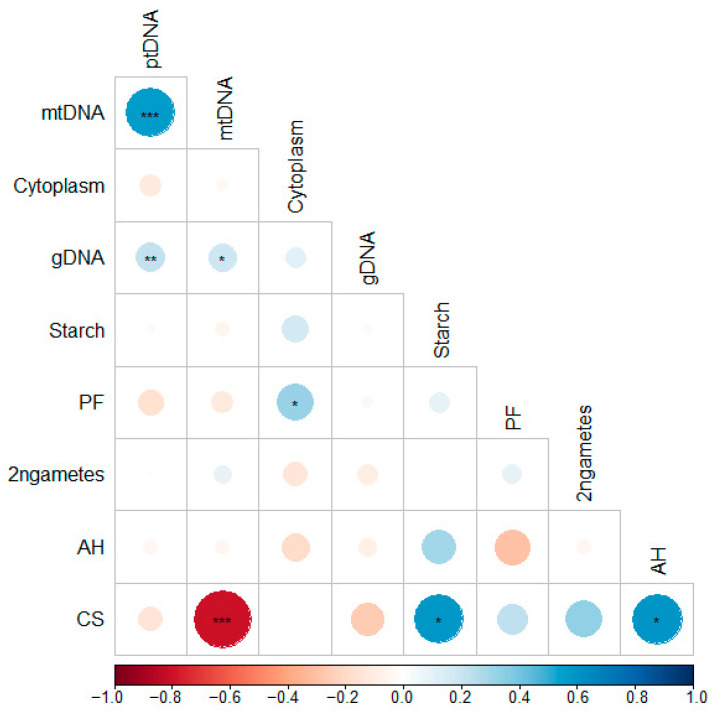
Kendall correlations among the tested traits. The circle size represents the correlation coefficient (R), while the colour indicates the direction (blue for positive, red for negative) and the strength of the correlation. The significance levels are (* *p* ≤ 0.05, ** *p* ≤ 0.01, *** *p* ≤ 0.001). ptDNA, plastid-to-nuclear DNA ratio; mtDNA, mitochondrial-to-nuclear DNA ratio; cytoplasm, type of cytoplasm; gDNA, nuclear DNA content; starch, tuber starch content; PF, pollen fertility; 2n gametes, presence of 2n gametes; AH, chip colour after harvest; CS, chip colour after 3 months of cold storage at 4 °C.

Kendall’s rank correlation analysis revealed a strong and statistically significant positive correlation between the plastid-to-nuclear DNA ratio (ptDNA) and the mitochondrial-to-nuclear DNA ratio (mtDNA) (*p* < 0.05; Figure 1).

Additionally, a significant positive correlation was observed between chip colour AH and CS (*p* < 0.05) and between starch content and CS (*p* < 0.05). A strong and significant negative correlation was found between CS and mtDNA content (*p* < 0.001). A multi-faceted relationship exists between starch content and CS. The storage of potato tubers at temperatures of approximately 4 °C has been demonstrated to induce a phenomenon referred to as cold-induced sweetening (CIS). In the context of such storage conditions, tuber starch undergoes a transformation into reducing sugars (glucose and fructose). These sugars, in turn, react with amino acids during the processing stage. This reaction has a deleterious effect on the quality of the potato product, manifesting in undesirable characteristics such as the onset of a dark chip colour [30]. Strach and reducing sugar contents are positively correlated and genotype-dependent [30,31]. We found that the pt/nucDNA ratio was higher for tubers characterized by a dark chip colour than for those with a light chip colour after cold storage, which is consistent with previous work [32]. Other significant associations included a moderate positive correlation between gDNA and ptDNA (*p* < 0.01) and weak but significant positive correlations between gDNA and mtDNA (*p* < 0.05) and between PF and cytoplasm type (*p* < 0.05). The correlation between the content of chloroplasts, mitochondria, and the nucleus arises from their distinct interdependent roles in cellular function and gene expression. Although each organelle retains its own genome and a high degree of independence, these three genomes function in close coordination. A nucleus partly controls plastid and mitochondrial genomes. A slow restructuring of these genomes has been observed, reflecting their evolutionary origins. Chloroplasts and mitochondria originated from ancient prokaryotes through endosymbiosis. Since then, much of their genetic material has been transferred to the nuclear genome, causing changes in its structure and size and provoking mutual dependence [33].

A relationship has between established between nucleus–cytoplasmic factors and the agronomic traits of potato, like black spot bruising, plant maturity, tuber shape, tuber yield, tuber starch content, chip quality, and resistance to late blight [19]. We observed no significant differences between cytoplasm type and chip quality traits (AH and CS). These results are consistent with the study by [19], where the correlation of different cytoplasm types with chip quality AH and CS indicated no significant differences.

**Figure 2:**
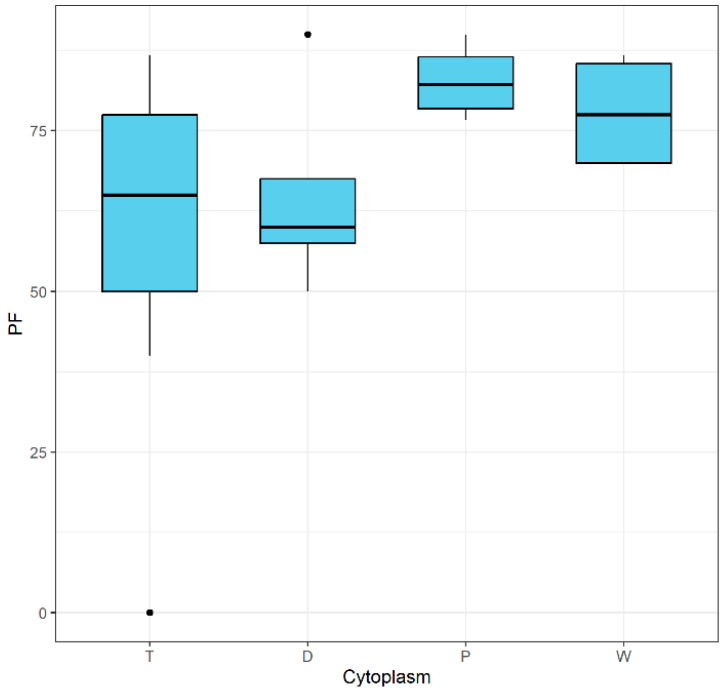
Boxplot showing pollen fertility (PF) in plants differing by cytoplasm type. The outliers are shown as black dots.

Although the differences in mean pollen fertility among genotypes of different cytoplasm types were not statistically significant (Table 3), no genotypes with pollen fertility below 50 were observed for cytoplasm types D, P, or W (Figure 2). In contrast, pollen fertility in genotypes with the T cytoplasm ranged from completely sterile (PF = 0) to 86.7%. Completely sterile genotype 90 HAE/35 with cytoplasm T is a dihaploid of *S. tuberosum*. Haploids derived from tetraploid potatoes frequently fail to flower and exhibit male sterility, primarily due to inbreeding depression associated with the haploidization process [34]. In group D, the mean pollen fertility was 65%, with only one plant being very fertile (PF = 90%), DG 00-849, whereas in plants with cytoplasm types P and W, the mean fertility was 82.8% and 77.9%, respectively (Figure 2). While the boxplot suggested that cytoplasms P and W tend to show higher and more stable pollen fertility, Tukey’s HSD test indicates that these differences are not statistically significant based on the current data. We observed a low correlation coefficient (R) between PF and cytoplasm types. We analyzed 30 diploid potato hybrids that possess cytoplasm types T, D, P, and W and produce fertile pollen grains with one exception: the individual with a T cytoplasm has completely infertile pollen. Cytoplasm type influences pollen fertility. Cytoplasmic male sterility (CMS) is an example of an agronomic trait, determined by the mutual interaction of the organellar and nuclear genomes. Potato cytoplasm types T, D, and W (W/γ) are associated with CMS, which manifests in different ways. T-type cytoplasm male sterility is characterized by the absence of pollen, no or poor pollen shedding, or deformities of the pollen and anthers. The W (W/γ)-type cytoplasm originating from *S. stoloniferum* causes tetrad–cytoplasmic male sterility. It is a specific male sterility, where pollen clusters in tetrads and is not functional. Potato genotypes with cytoplasm D, derived from *S. demissum*, produce abundant and stainable pollen (viable and fertile), but it is not effective in crosses with *S. tuberosum* [18,35].

Genotypes with a cytoplasm type suggesting CMS does not always exhibit this trait, because fertility can be restored in the presence of nuclear Rf genes, encoding fertility restorer proteins [36]. Fertility restorers in potato have not been fully identified. Reference [37] proposed the pentatricopeptide repeat proteins as Rf genes in potato. PPR proteins act directly on mitochondrial transcripts and influence the development of the CMS trait [38]. Identifying fertility restorers and developing molecular markers based on their sequence will enable the selection of desirable individuals and the matching of parental forms in breeding programmes, even in crosses with CMS lines.

**Figure 3:**
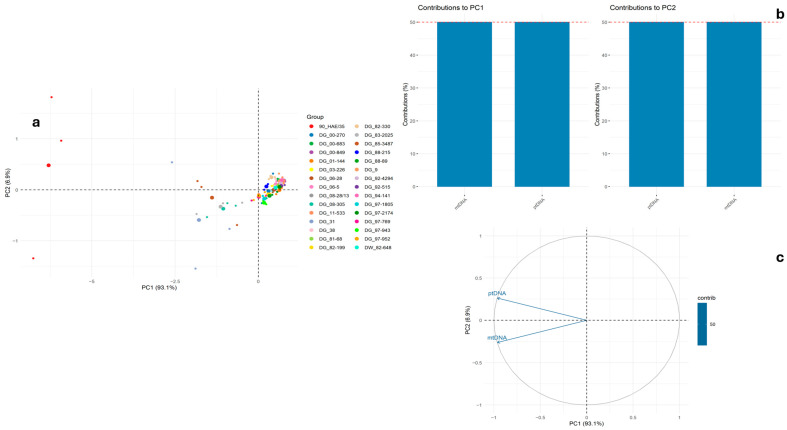
Results of PCA for mean (from three technical replicates) of mitochondria and plastid content, showing relationships among samples (**a**) and contributions of each variable to PC1 and PC2 (**b**), and (**c**) variable correlation plot (PC1 vs. PC2). The large dot represents the mean for each group (genotype). The red line represents the expected average contribution of a variable if all variables contribute equally.

PCA revealed that the first principal component (PC1) explains the vast majority of variation in the dataset (93.1%), primarily driven by ptDNA and mtDNA variation (Figure 3a). The majority of genotypes clustered tightly around the origin, indicating overall similarity in the analyzed traits. The PCA results indicate that variation in ptDNA and mtDNA content is the primary driver of the first principal component (PC1) and are consistent with previous studies highlighting the significant role of organellar genomes in differentiating plant taxa, particularly in the context of environmental adaptation and evolutionary history [39]. Many plant species exhibit strong correlations between plastid and mitochondrial variation, due to their common maternal inheritance.

In contrast, a small number of genotypes, particularly the 90 HAE/35, exhibited marked separation along PC1, reflecting distinct levels of ptDNA and mtDNA (Figure 3a). It is proof that organellar DNA can serve as an effective marker of population differentiation. Both ptDNA and mtDNA contributed approximately equally to PC1 and PC2 (~50%) (Figure 3b). In the PCA biplot (Figure 3c), the vectors for ptDNA and mtDNA pointed in similar directions along the PC1 axis, indicating a strong correlation between the two variables and their dominant contribution to the total variance.

**Figure 4:**
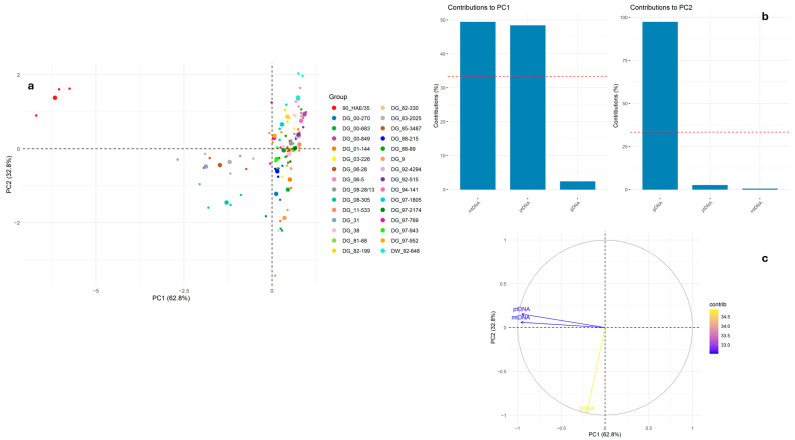
Results of PCA for mean (from three technical replicates) of mitochondria, plastids, and gDNA, showing relationships among samples (**a**), contributions of each variable with PC1 and PC2 (**b**), and (**c**) variable correlation plot (PC1 vs. PC2). The large dot represents the mean for each group (genotype). The red line represents the expected average contribution of a variable if all variables contribute equally.

Subsequent PCA based on additional variables showed that PC1 and PC2 together accounted for approximately 95% of the total variance (Figure 4a). The primary source of variation along PC1 was again attributed to ptDNA and mtDNA, whereas PC2 reflected variation primarily explained by gDNA. Genotype 90 HAE/35 was clearly distinct, likely due to substantially different gDNA levels (Figure 4a). These findings confirm that ptDNA and mtDNA variation drives PC1, while PC2 is influenced predominantly by gDNA (Figure 4b). The PCA biplot further supported the strong positive correlation between ptDNA and mtDNA, while gDNA appeared to be largely independent from these variables (Figure 4c).

**Figure 5:**
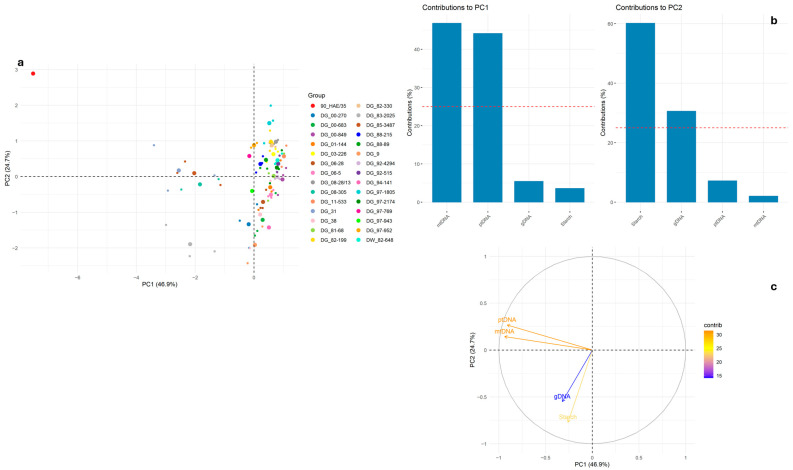
Results of PCA for mean (from three technical replicates) of mitochondria, plastids, gDNA, and starch content, showing relationships among samples (**a**), contributions of each variable to PC1 and PC2 (**b**), and (**c**) variable correlation plot (PC1 vs. PC2). The large dot represents the mean for each group (genotype). The red line represents the expected average contribution of a variable if all variables contribute equally.

In a subsequent PCA including starch content, PC1 (46.9%) and PC2 (24.7%) collectively explained nearly 72% of the total variance (Figure 5a). Most genotypes clustered centrally, except the genotype 90 HAE/35, which again showed distinct values, indicative of divergence in one or more variables (Figure 5a). ptDNA and mtDNA were the strongest contributors to PC1 (~45% each), while starch content was the major contributor to PC2 (~60%), with gDNA contributing to a lesser extent (Figure 5b). ptDNA and mtDNA exhibited a positive correlation and aligned along PC1. In contrast, gDNA and starch showed limited association with ptDNA and mtDNA (Figure 5c).

The dry matter and starch content in tubers was positively correlated with both the plastid-to-nuclear DNA ratio (pt/nuc DNA) and the mitochondrial-to-nuclear DNA ratio (mt/nuc DNA). This means that higher proportions of these organelles (measured by DNA content) are correlated with higher starch production in potato tubers [40], indicating that increased starch synthesis requires increased expression of related genes, organelle genome templates, and organelle numbers per cell. The nuclear genes associated with starch metabolism are known [31]. Starch biosynthesis in plants is also linked to both the efficiency of photosynthesis in chloroplasts and the functioning of mitochondria. The exact mechanism of this interdependence between these genomes in the context of starch synthesis is unknown. It is assumed that these organelles communicate with each other through signalling factors and mutually regulate the expression of genes related to starch metabolism [6,41].

The identification of candidate genes and cytoplasm-mediated regulatory factors that influence starch content and quality will enable the development of genetic markers. It will also allow for better selection of parental components in the breeding of potato varieties with increased or decreased starch content and specific starch parameters.

## 3. Materials and Methods

### 3.1. Plant Material

The research material consists of 30 diploid interspecific potato hybrids: DG 82-199; DG 81-68; DG 92-4294; DG 92-515; DG 88-215; DG 88-89; DG 97-943; DG 97-769; DG 08-28/13; DG 97-952; DG 97-2174; DG 01-144; DG 08-305; DG 38; DG 31; DG 85-3487; DG 83-2025; DG 00-270; DG 00-683; DG 06-5; DG 00-849; DG 06-28; DG 94-141; DG 03-226; DG 82-330; DG 11-533; DG 9; DG 97-1805; 90 HAE/35; and DW 82-648 constitute part of the diploid potato collection at IHAR-PIB, Młochów Division (POL047). They were obtained by recombinant breeding in the years 1980–2011, through introgression of genetic material from wild and primitively cultivated potato species into an *S. tuberosum* background. They possess *S. chacoense*, *S. phureja*, *S. yungasense*, *S.gourlayi*, *S. microdontum*, *S. verrucosum*, *S. acaule*, *S. stenotomum*, *S. demissum*, and *S. stoloniferum* in their pedigree. The composition of 30 diploid interspecific potato hybrids is presented in Supplementary Table S3. They were bred in various breeding directions: producing 2n gametes, chipping quality, resistance to *P. infestans*, Pectobacteria and potato viruses, high starch content, and good taste.

To estimate genome size, organelle content, and cytoplasm type, plants were maintained in 2024 in vitro culture conditions on standard MS medium supplemented with 3% sucrose and solidified with 0.8% agar, with pH 5.7, cultivated at 20 °C with 16 h illumination. To analyze genome size stability under varying cultivation conditions, three randomly chosen diploid interspecific potato hybrids were utilized: DG 08-28/13, DG 00-683, and DG 08-305. These genotypes grew in 2024 under distinct conditions: in vitro and in a greenhouse. The plants were propagated from in vitro (post-in vitro planting) into pots. The first tuber generation was obtained from these plants and subsequently planted in the greenhouse in 2025.

### 3.2. Sample Preparation for Flow Cytometry (FCM)

The general methodology of FCM was based on that in [42]. Preliminary analyses included determining the appropriate amount of sample and standard tissue needed to obtain sufficient nucleus count and good quality peaks. For potato diploid hybrids, approx. 60 mg of fresh leaves was used to prepare the sample, and for *Raphanus sativus* (L.) cv. Saxa, 40 mg of fresh leaves was used. The potato and radish young leaves were chopped together in 1.5 mL of the cold general-purpose buffer (GPB) on ice with a razor blade to isolate nuclei. The suspension was passed through a mesh filter (30 µm) into 2 mL sample tubes. To 1 mL of nuclei suspension, RNase A (50 µg·mL^−1^) and propidium iodide (PI) at 50 µg·mL^−1^ were added. The sample was incubated on ice for 10 min in the dark.

### 3.3. Genome Size Estimation

For genome size estimation, three plants were analyzed in three replicates on three days (nine samples) for each potato diploid hybrid. Samples were analyzed using the CyFlow Space flow cytometer (Sysmex Partec GmbH, Görlitz, Germany) equipped with a blue laser (488 nm) followed by instrument calibration using 3 µm calibration beads (Sysmex, no. 05-4018). For each sample 3000 nuclei were counted. For each histogram, the coefficient of variance (CV) was recorded for the G1 peak of the sample and the standard. The nuclear genome size of the diploid potato hybrids was estimated using *R. sativus* cv. Saxa (2C-value = 1.11 pg) as the reference standard [43]. The 2C nuclear DNA content was calculated according to the following formula: (sample G0/G1 mean fluorescence/reference standard G0/G1 mean fluorescence) × 2C nuclear DNA content of reference standard. The genome size was expressed as 2C-value (pg) and in Mbp, determined according to the conversion 1 pg = 978 Mbp [44].

### 3.4. DNA Extraction

Total DNA was extracted from 3 independent plants per genotype, from 200 mg of the upper parts of 4–6-week-old in vitro plants using a DNeasy Plant Mini Kit (Qiagen, Hilden, Germany), according to the manufacturer’s protocol. DNA concentration and quality were determined using a NanoDrop spectrophotometer (Thermo Fisher Scientific, Waltham, MA, USA). The integrity of DNA was checked on an agarose gel. For analysis, the DNA concentration was adjusted to 20 ng/µL.

### 3.5. qPCR for Determination of Organelle DNA Content

Determination of organelle DNA content was performed according to [41]. The relative quantification of plastid DNA (ptDNA) and mitochondrial DNA (mtDNA) in comparison with nuclear DNA (nucDNA) was performed using the Lightcycler 480 II System (Roche, Basel, Switzerland). The PCR conditions were 95 °C for 3 min followed by 40 cycles of 95 °C for 10 s, 60 °C for 30 s, and 72 °C for 30 s. The PCR primer pairs for ptDNA and nucDNA were used according to [41]. The primers were designed for the single-copy region of plastid DNA (5′-TCCGACAACTGGTGGAGTGACAG-3′; 5′-TGCTTGTGAACCTTCGGGTAC-3′), for mitochondrial DNA (5′-GGTCCGATGGCTGTTCTCCAC-3′; 5′-CAGTACTGAGAAGCATGTGCCCAT-3′), and for endogenous nuclear genome control (5′-TACAAGGCCAAAGTTAAGAAAGCA-3′; 5′-AGATTGAGGAAGAAACATCTCCCAT-3′) [41]. Based on the obtained results, the pt/nuc ratio and mt/nuc ratio were obtained.

### 3.6. Determination of Cytoplasm Type

For cytoplasm type determination, multiplex PCR reaction amplification with four markers specific to cpDNA (T, S, SAC, and A) and one specific to mtDNA (D) was applied. Determination of cytoplasm type was performed according to [18] with modifications described in [45,46].

### 3.7. Determination of Agronomic Traits

Total starch content (TSC, percent of fresh weight) was calculated based on the ratio of tuber weight in the air to tuber weight in the water, following the method described by [47]. Pollen fertility (PF; percentage of Lactofuchsin-stained pollen grains) and the presence of 2n gametes, defined as the percentage of grains stained with Lactofuchsin, were evaluated using an indirect staining technique described by [48]. Pollen was collected from three flowers per plant and placed directly onto microscope slides. A drop of lactofuchsin solution (20 mL each of phenol and lactic acid, 40 mL of glycerine, and 8 mL 1% solution of fuchsin in water) was applied, and the round, stained pollen grains were immediately counted under the microscope across ten fields of view. Pollen with at least 30% of Lactofuchsin-stained pollen grains was classified as fertile. Among the pollen grains, large and distinct grains corresponding to unreduced (2n) gametes were identified. Chip colour after harvest (AH) and after 3 months of cold storage at 4 °C (CS) was evaluated according to [49]. Tubers from both parental and progeny plants were evaluated for chip colour immediately after harvest (AH) and following 3 months of cold storage at 4 °C (CS). For each genotype, frying was conducted in three replications. In each replication, four slices were prepared from each of two tubers. Four slices, approximately 1 mm in thickness, were obtained from each tuber and fried in oil at 180 °C for 2–3 min. The colour of each slice was assessed visually using a 9-point scale, where 1 represents very dark and 9 represents very light, according to [49]. The phenotypic data of the traits studied in this paper can be found in [29].

### 3.8. Statistical Analysis

The distribution of the data was assessed using the Shapiro–Wilk test, and the difference among mean plant fertility values in each cytoplasm group was tested with ANOVA and Tukey’s HSD in R version 4.2.2 [50]. Kendall’s rank correlation was calculated and visualized using the corrplot v. 0.95 R package [51] and the ggpubr v. 0.6.0 R package [52]. Principal component analysis (PCA) was performed using the base stats R package and visualized with the factoextrav.1.0.7 R package [53].

## 4. Conclusions

A number of agronomic traits are taken into account in the breeding process for new potato varieties. The results of our research highlight that organellar genome composition, alongside nuclear DNA, plays a crucial role in shaping important agronomic traits. This knowledge offers valuable insights for potato breeding programmes, suggesting the targeted selection of cytoplasmic types in combination with nuclear markers to obtain the most suitable organelle–nuclear genome combinations to enhance breeding efficiency, particularly for traits like starch content, male fertility, and processing quality. Diploids interspecific hybrids are suitable material, carrying new sources of important agronomic traits for potato breeding.

## Figures and Tables

**Table 1:** Estimation of genome size in diploid potato hybrids cultivated in vitro, after post-in vitro planting, and grown from first tuber generation.

Potato Diploid Hybrid	Growth Conditions	2C-Value ^1^ (pg)	±SD	Peak CV ^2^ (%) Sample	Peak CV (%) Standard
DG 08-28/13	in vitro	1.739 ns	0.024	3.12	3.42
	post-in vitro planting	1.778 ns	0.034	3.06	3.18
	1st tuber generation	1.770 ns	0.035	2.96	3.14
DG 00-683	in vitro	1.797 ns	0.077	3.36	3.15
	post-in vitro planting	1.747 ns	0.039	3.69	3.25
	1st tuber generation	1.751 ns	0.011	4.12	3.65
DG 08-305	in vitro	1.746 ns	0.059	2.97	3.05
	post-in vitro planting	1.720 ns	0.015	3.02	3.54
	1st tuber generation	1.723 ns	0.011	3.54	4.04

^1^ mean 2C-value, ^2^ coefficient variation for G1/G0 peak, ns—not statistically different (one-way ANOVA test, *p* = 0.05) within each potato diploid hybrid, letters shared indicated no significant differences in mean 2C-value (Tukey’s HSD test, *p* = 0.05); each value is the mean of nine replicates; and *Raphanus sativus* cv. Saxa (2C = 1.1 pg) was applied as an internal standard.

**Table 2:** Results of Shapiro–Wilk normality test.

Trait	W	*p* *
ptDNA	0.42752	2.2 × 10^−16^
mtDNA	0.61772	5.844 × 10^−14^
Cytoplasm	0.67698	9.025 × 10^−13^
gDNA	0.96597	0.01852
Starch	0.96254	0.0258
PF	0.88719	0.00297
2n gametes	0.60373	3.266 × 10^−7^
AH	0.66192	1.386 × 10^−5^
CS	0.88965	0.1377

* The significance level: *p* < 0.05. ptDNA, plastid-to-nuclear DNA ratio; mtDNA, mitochondrial-to-nuclear DNA ratio; Cytoplasm, type of cytoplasm; gDNA, nuclear DNA content; Starch, tuber starch content; PF, pollen fertility; 2n gametes, presence of 2n gametes; AH, chip colour after harvest; CS, chip colour after 3 months of cold storage at 4 °C.

**Table 3:** Results of Tukey’s HSD post hoc test comparing means of pollen fertility (PF) among different cytoplasm types.

Cytoplasm	Diff	Lwr	Upr	*p* Adj
D-T	4.25	−23.6377	32.13765	0.975263
P-T	22	−5.88765	49.88765	0.161192
W-T	17.16667	−10.721	45.05432	0.352392
P-D	17.75	−18.2528	53.75281	0.542416
W-D	12.91667	−23.0861	48.91947	0.762085
W-P	−4.83333	−40.8361	31.16947	0.982821

## Data Availability

The original contributions presented in this study are included in the article/Supplementary Materials. Further inquiries can be directed to the corresponding author.

## Supplementary Materials

The following supporting information can be downloaded at: https://www.mdpi.com/article/10.3390/ijms262210841/s1.
