# Supplementary material for: Cytoplasmic and Nuclear Effects on Agronomic Traits in Diploid Interspecific Potato Hybrids
Source: Int J Mol Sci. 2025 Nov 8;26(22):10841. doi: 10.3390/ijms262210841 (PMC12652364; doi:10.3390/ijms262210841)
Supplement: Supplementary file 1 [file ijms-26-10841-s001.zip › Supplementary Table S1.pdf]

Supplementary Table S1. Estimation of genome size in diploid potato hybrids.

Potato diploid hybrid	2C-value[1] (pg)	±SD	Peak CV[2] (%) sample	Peak CV (%) standard	Diploid genome size (Gbp)
DG 9	1.833a	0.062	4.20	3.98	1.792
DG 08-305	1.824ab	0.020	3.24	3.19	1.784
DG 00-270	1.804a-c	0.033	4.05	3.94	1.765
DG 00-683	1.797a-d	0.077	3.12	3.40	1.757
DG 94-141	1.709f-j	0.027	3.33	3.31	1.671
DG 31	1.783a-e	0.027	4.28	4.11	1.743
DG 85-3487	1.779a-e	0.014	3.49	3.23	1.740
DG 88-215	1.776a-f	0.013	2.77	2.87	1.737
DG 83-2025	1.773a-g	0.024	3.39	3.12	1.734
DG 97-943	1.760b-h	0.045	3.25	3.09	1.721
DG 38	1.752c-h	0.104	2.15	2.09	1.713
DG 88-89	1.748c-i	0.039	2.81	2.52	1.710
DG 82-330	1.748c-i	0.011	3.72	3.18	1.710
DG 06-28	1.748c-j	0.023	3.95	3.19	1.709
DG 03-226	1.748c-j	0.038	3.69	3.04	1.709
DG 81-68	1.747c-j	0.044	3.24	2.99	1.708
DG 97-2174	1.744c-j	0.020	2.90	3.12	1.706
DG 08-28/13	1.739c-j	0.024	2.42	2.99	1.700
DG 11-533	1.739c-j	0.011	2.96	3.09	1.700
DG 97-769	1.735d-j	0.050	2.72	2.40	1.696
DG 97-952	1.732d-j	0.011	3.91	3.21	1.694
90 HAE/35	1.731d-j	0.024	4.24	4.02	1.693
DG 92-4294	1.729e-j	0.012	2.95	2.35	1.691
DG 92-515	1.727e-j	0.010	2.77	2.04	1.689
DG 97-1805	1.716e-j	0.026	3.92	3.78	1.679
DG 94-141	1.709f-j	0.027	3.33	3.31	1.671
DG 82-199	1.706g-j	0.009	2.25	2.29	1.668
DG 00-849	1.700h-j	0.012	3.88	3.65	1.663
DG 06-5	1.682ij	0.022	3.22	3.02	1.645
DW 82-648	1.680j	0.047	2.95	2.77	1.643

[1] mean 2C-value, [2] coefficient variation for G1/G0 peak, letters shared indicated no significant differences in mean 2C-value (Tukey's HSD test, P = 0.05), each value is the mean of nine replicates, *Raphanus sativus* cv. Saxa (2C = 1.1 pg) was applied as an internal standard
