# Supplementary material for: Cytoplasmic and Nuclear Effects on Agronomic Traits in Diploid Interspecific Potato Hybrids
Source: Int J Mol Sci. 2025 Nov 8;26(22):10841. doi: 10.3390/ijms262210841 (PMC12652364; doi:10.3390/ijms262210841)
Supplement: Supplementary file 1 [file ijms-26-10841-s001.zip › Supplementary Table S2.pdf]

Supplementary Table S2. Analysis of mitochondrial and plastid content, and cytoplasm types of 30 diploid interspecific potato hybrids.

Genotype	Mean pt/nuc DNA	Mean mt/nuc DNA	Cytoplasm type
DG 82-199	524.58	92.59	P
DG 81-68	342.24	44.36	T
DG 92-4294	312.10	32.39	T
DG 92-515	217.06	36.41	D
DG 88-215	810.79	82.34	T
DG 88-89	434.61	92.01	T
DG 97-943	465.19	121.86	T
DG 97-769	846.84	125.93	T
DG 08-28/13	393.74	58.71	D
DG 97-952	875.26	123.31	T
DG 97-2174	257.09	43.47	P
DG 01-144	176.71	50.15	D
DG 08-305	1994.37	257.77	T
DG 38	449.46	55.99	P
DG 31	2696.74	354.16	W
DG 85-3487	2759.98	270.33	T
DG 83-2025	2162.50	262.07	W
DG 00-270	602.58	85.49	D
DG 00-683	459.51	32.10	T
DG 06-5	480.50	48.97	T
DG 00-849	245.30	17.43	D
DG 06-28	410.34	66.08	T
DG 94-141	346.17	23.14	T
DG 03-226	495.77	57.10	T
DG 82-330	850.68	49.22	T
DG 11-533	267.14	19.49	T
DG 9	407.54	31.44	W
DG 97-1805	569.69	113.27	T
90 HAE/35	10384.76	702.74	T
DW 82-648	397.27	56.46	T
