# Supplementary material for: Cytoplasmic and Nuclear Effects on Agronomic Traits in Diploid Interspecific Potato Hybrids
Source: Int J Mol Sci. 2025 Nov 8;26(22):10841. doi: 10.3390/ijms262210841 (PMC12652364; doi:10.3390/ijms262210841)
Supplement: Supplementary file 1 [file ijms-26-10841-s001.zip › Supplementary Table S3.pdf]

Supplementary Table 3. Composition of 30 diploid interspecific potato hybrids.

**Diploid interspecific potato hybrids**	**Composition of diploid hybrids**
DG 82-199	*chc, phu, tbr, yun*
DG 81-68	*chc, tbr, yun*
DG 92-4294	*chc, grl, phu, stn, tbr, yun*
DG 92-515	*tbr, grl, chc, yun, phu, ver, mcd*
DG 88-215	*chc, grl, mcd, tbr, ver, yun*
DG 88-89	*ter, chc, grl, yun*
DG 97-943	*acl, chc, dms, grl, phu, stn, sto, tbr, yun*
DG 97-769	*acl, chc, dms, grl, phu, stn, sto, tbr, yun*
DG 08-28/13	*acl chc dms grl mcd phu stn sto tbr ver yun*
DG 97-952	*acl, chc, dms, grl, phu, stn, sto, tbr, yun*
DG 97-2174	*acl chc dms grl phu stn sto tbr yun*
DG 01-144	*chc grl mcd phu stn tbr ver yun*
DG 08-305	*acl chc dms grl mcd phu stn tbr ver yun*
DG 38	*mcd, tbr, ver*
DG 31	*chc, mcd, tbr, yun*
DG 85-3487	*chc, mcd, tbr, ver*
DG 83-2025	*chc, tbr, yun*
DG 00-270	*tbr chc mcd phu ver yun*
DG 00-683	*tbr, grl, chc, ver, mcd, yun*
DG 06-5	*acl chc dms grl mcd phu sto tbr ver yun*
DG 00-849	*acl, chc, dms, grl, mcd, phu, stn, sto, tbr, ver, yun,*
DG 06-28	*acl, chc, dms, grl, mcd, phu, sto, tbr, ver, yun*
DG 94-141	*tbr, chc, yun, ver, mcd, grl*
DG 03-226	*acl chc dms grl mcd phu sto stn tbr ver yun*
DG 82-330	*tbr, chc,yun,phu*
DG 11-533	*acl chc dms grl mcd phu stn sto tbr ver yun*
DG 9	*chc, tbr, yun*
DG 97-1805	*acl chc dms grl mcd phu sto tbr ver yun*
90 HAE/35	*tbr*
DW 82-648	*chc, grl, tbr*

S. chacoense (chc), *S. phureja (phu)*, *S. yungasense (yun)*, *S.gourlayi (grl)*, *S. microdontum (mcd)*, *S. verrucosum (ver)*, *S. acaule (acl)*, *S. stenotomum (stn)*, *S. demissum* (*dms*) and *S. stoloniferum (sto), S. tuberosum (tbr)*
